# Ground-glass nodules and CT-guided placement of platinum coils

**DOI:** 10.1590/S1806-37132014000400015

**Published:** 2014

**Authors:** Bruno Hochhegger, Fabíola Adélia Perin, Spencer Marcantonio Camargo, Edson Marchiori, Klaus Irion, Marcos Duarte Guimarães, Jose Carlos Felicetti, Jose Camargo

**Affiliations:** Santa Casa Hospital Complex in Porto Alegre; and Professor of Radiology, Federal University of Health Sciences of Porto Alegre, Porto Alegre, Brazil; Santa Casa Hospital Complex in Porto Alegre, Porto Alegre, Brazil; Santa Casa Hospital Complex in Porto Alegre, Porto Alegre, Brazil; Federal University of Rio de Janeiro, Rio de Janeiro, Brazil; Liverpool Heart and Chest Hospital, and Royal Liverpool and Broadgreen University Hospital, Liverpool, United Kingdom; A.C. Camargo Cancer Center, São Paulo, Brazil; Santa Casa Hospital Complex in Porto Alegre; and Professor of Surgery, Federal University of Health Sciences of Porto Alegre, Porto Alegre, Brazil; Santa Casa Hospital Complex in Porto Alegre; and Professor of Surgery, Federal University of Health Sciences of Porto Alegre, Porto Alegre, Brazil

## To the Editor:

The detection of a small growing pulmonary nodule on chest CT raises the suspicion of lung cancer, but proof of malignancy must be established by either needle biopsy or nodule resection.^(^ 1 ^)^ Pulmonary nodules ≤ 10 mm with ground-glass opacity should be considered to have a high possibility of malignancy.^(^ 2 ^)^ Various centers perform the excision of these small growing nodules using video-assisted thoracoscopic surgery (VATS) in order to minimize postoperative morbidity, as well as to remove as small a volume of lung tissue as possible. Small nodules are often visible with the thoracoscope if they lie within 5 mm of the visceral pleural surface; however, if they are located deeper in the lung, palpation is required in order to locate them for excision. A previous study found that, in a series of 92 consecutive patients undergoing VATS, 50 (54%) required conversion to thoracotomy.^(^ 3 ^)^ The most common reason for conversion to full thoracotomy was failure to locate the nodule. Univariate and multivariate analysis of the eleven variables studied showed that if the distance from the pleural surface to the nodule edge was greater than 5 mm, the probability of failure to detect a nodule was 63%,^(^ 3 ^)^ and 40% of those nodules were found to be malignant. Because of the difficulty in localizing a nodule during surgery and the increasing clinical load due to the identification of small lung nodules for lung cancer screening using CT, there has been extensive investigation for improving nodule localization techniques in order to assist the resection of small nodules during VATS. We would like to report the first use of a new technique for the intraoperative localization of such nodules in Brazil: CT-guided placement of platinum coils.

**Figure 1:**
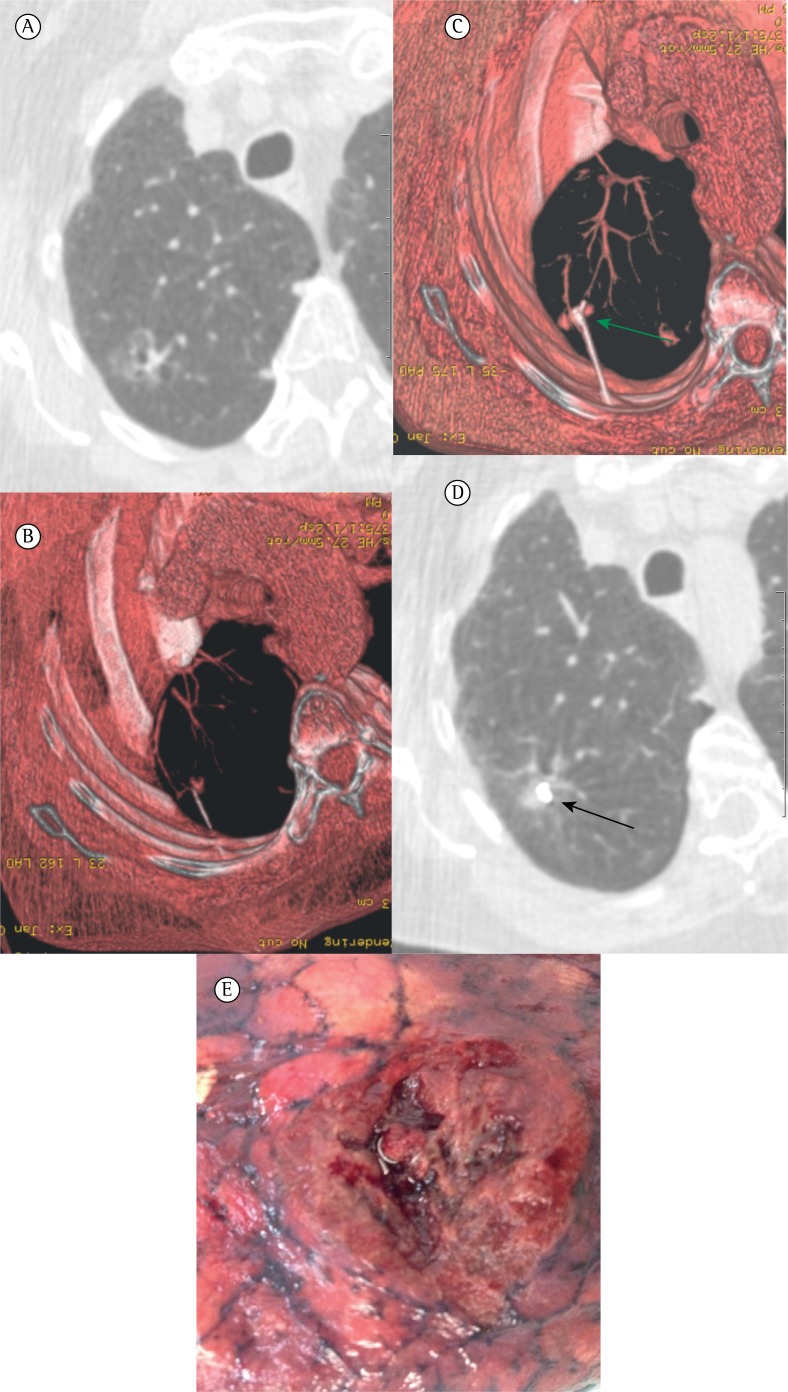
In A, a CT scan demonstrating a 1-cm ground-glass nodule in the central portion of the right upper lobe. In B, volume rendering of a CT scan demonstrating the needle inside the ground-glass nodule in right upper lobe. In C, volume rendering of a CT scan demonstrating the CT-guided placement (arrow) of a platinum microcoil inside the ground-glass nodule. In D, a CT scan taken after the procedure, demonstrating the platinum coil (arrow) inside the ground-glass nodule. In E, a photograph of the surgical specimen showing the coil.

A 72-year-old woman underwent a chest CT for the evaluation of chronic cough. The CT scans demonstrated a 1-cm ground-glass nodule in the central portion of the right upper lobe (Figure 1A). The nodule was later biopsied, and the final pathological examination revealed atypical cells suspected of being adenocarcinoma *in situ* (formerly known as bronchioalveolar carcinoma). Surgical resection using VATS was planned; however, because of the ground-glass nature of the nodule and its distance from the pleural surface, preoperative wire localization was requested. Using CT guidance, the tip of the loaded Chiba needle was percutaneously placed approximately 5 mm deep into the lung nodule (Figure 1B). The guide wire was introduced up to the first mark, advancing 30 mm of the fiber-coated coil out of the Chiba needle and into the lung parenchyma, where it assumed a tightly coiled helical configuration into the nodule (Figures 1C and 1D). The patient underwent VATS, and the coil was easily localized by lung palpation through a 3-cm minithoracotomy (Figure 1E). The final diagnosis was pulmonary adenocarcinoma.

Techniques for the localization of pulmonary nodules have been classified into three types. ^(^ 1 ^,^ 4 ^)^ The first class uses intraoperative imaging (either ultrasonography or CT). Localization with intraoperative ultrasound is difficult because the lung must be completely collapsed in order to allow the visualization of small nodules.^(^ 1 ^,^ 4 ^)^ This technique lengthens the surgical time, since the complete collapse of the lung can take 30-150 min and is often contraindicated in patients with extensive emphysema. Not only is experience with real-time CT-guided thoracoscopic resection limited, but also artifacts caused by instruments and staples degrade the CT image, and the limited space within the scanner gantry makes the procedure difficult.^(^ 1 ^,^ 4 ^)^

The second class of targeting techniques includes the percutaneous injection of dyes, contrast media, radionuclides, or colored adhesive agents.^(^ 1 ^,^ 4 ^,^ 5 ^)^ Diffusion away from the nodule is a limitation of these techniques and imposes restrictions on the allowable time between the CT localization procedure and the thoracoscopic resection. This can cause difficulties in the operating room scheduling. In addition, certain dyes, such as methylene blue, carry a possible risk of anaphylactic reactions following their injection and are often difficult to visualize on the visceral pleural surface in patients with extensive anthracotic pigmentation of the lungs. ^(^ 1 ^,^ 4 ^,^ 5 ^)^ Because these materials are not water-soluble, they carry a potential risk of stroke if they gain access to the pulmonary veins.

The third class of targeting techniques uses coils or microcoils that are soft and pliable and cause little damage to lung tissue, even when dislodged. A previous study compared the use of microcoils and hook wires for the localization of nodules in freshly harvested goat lungs.^(^ 5 ^)^ The authors reported that when a coil was displaced, it would uncoil, causing minimal tissue damage. In addition, the "fuzzy" fiber coating on these microcoils induces coagulation and increases the adhesion of the coil to the lung tissue. The coiled configuration and the fiber coating virtually eliminate the risk of embolization.

In conclusion, we would like to highlight this new method of nodule localization, which is a safe and effective technique and increases the success rate of nodule excision using VATS, especially for small, ground-glass nodules.
