# Supplementary material for: Ground-glass nodules and CT-guided placement of platinum coils
Source: J Bras Pneumol. 2014 Jul-Aug;40(4):450–2. doi: 10.1590/S1806-37132014000400015 (PMC4201178; doi:10.1590/S1806-37132014000400015)
Supplement: Translation

# Translation: Nódulos em vidro fosco e marcadores espirais de platina guiados por TC

**Authors:** Bruno Hochhegger, Fabíola Adélia Perin, Spencer Marcantonio Camargo, Edson Marchiori, Klaus Irion, Marcos Duarte Guimarães, Jose Carlos Felicetti, Jose Camargo

**Affiliations:** Complexo Hospitalar Santa Casa de Porto Alegre, e Professor de Radiologia, Universidade Federal de Ciências da Saúde de Porto Alegre, Porto Alegre (RS) Brasil; Complexo Hospitalar Santa Casa de Porto Alegre, Porto Alegre (RS) Brasil; Complexo Hospitalar Santa Casa de Porto Alegre, Porto Alegre (RS) Brasil; Universidade Federal do Rio de Janeiro, Rio de Janeiro (RJ) Brasil; Liverpool Heart and Chest Hospital, e Royal Liverpool and Broadgreen University Hospital, Liverpool, Reino Unido; A.C. Camargo Cancer Center, São Paulo (SP) Brasil; Complexo Hospitalar Santa Casa de Porto Alegre, e Professor de Cirurgia, Universidade Federal de Ciências da Saúde de Porto Alegre, Porto Alegre (RS) Brasil; Complexo Hospitalar Santa Casa de Porto Alegre, e Professor de Cirurgia, Universidade Federal de Ciências da Saúde de Porto Alegre, Porto Alegre (RS) Brasil

## Ao Editor:

Embora a detecção de um pequeno nódulo pulmonar crescente na TC de tórax levante a suspeita de câncer de pulmão, a malignidade se comprova por meio de biópsia com agulha ou resseção do nódulo.^(^ 1 ^)^ Deve-se considerar que a possibilidade de malignidade é grande em nódulos pulmonares ≤ 10 mm com opacidade em vidro fosco.^(^ 2 ^)^ Em vários centros, a excisão desses pequenos nódulos crescentes é feita por meio de videotoracoscopia, a fim de minimizar a morbidade pós-operatória e remover o menor volume possível de tecido pulmonar. Nódulos pequenos podem ser vistos por meio de um toracoscópio se estiverem a 5 mm da superfície pleural visceral; no entanto, se estiverem mais fundos no pulmão, a palpação é necessária a fim de localizá-los para a excisão. Em uma série de 92 pacientes consecutivos submetidos a videotoracoscopia, foi necessário converter o procedimento em toracotomia em 50 (54%). ^(^ 3 ^)^ O motivo mais comum da conversão da videotoracoscopia em toracotomia completa foi a impossibilidade de localizar o nódulo. A análise univariada e multivariada das onze variáveis estudadas mostrou que a probabilidade de não detecção de um nódulo era de 63% se a distância entre a superfície pleural e a margem do nódulo fosse maior que 5 mm,^(^ 3 ^)^ e 40% dos nódulos revelaram-se malignos. Por causa da dificuldade em localizar um nódulo durante a cirurgia e da carga clínica cada vez maior em virtude do uso de TC para identificação de pequenos nódulos pulmonares no rastreio de câncer de pulmão, tem havido extensa investigação para melhorar as técnicas de localização de nódulos, a fim de auxiliar a resseção de pequenos nódulos durante a videotoracoscopia. Gostaríamos de relatar o primeiro uso no Brasil de uma nova técnica para a localização intraoperatória de tais nódulos: a inserção de micromolas de platina guiada por TC. 

Uma mulher de 72 anos com tosse crônica foi submetida a TC de tórax para avaliação. As imagens revelaram um nódulo de 1 cm em vidro fosco na porção central do lobo superior direito (Figura 1A). O nódulo foi posteriormente submetido a biópsia, e o exame anatomopatológico final revelou células atípicas que levantaram a suspeita de adenocarcinoma in situ (anteriormente denominado carcinoma bronquioalveolar). Planejou-se a resseção cirúrgica por meio de videotoracoscopia; no entanto, como se tratava de um nódulo em vidro fosco - e em virtude da distância entre o nódulo e a superfície pleural - solicitou-se a localização pré-operatória com fio guia. Por meio de orientação tomográfica, a ponta da agulha de Chiba carregada foi inserida no nódulo pulmonar, por via percutânea, até a profundidade de aproximadamente 5 mm (Figura 1B). O fio guia foi introduzido até a primeira marca, empurrando 30 mm da micromola revestida de fibra para fora da agulha de Chiba e para dentro do parênquima pulmonar, onde assumiu uma configuração helicoidal firmemente enrolada dentro do nódulo (Figuras 1C e 1D). A paciente foi submetida a videotoracoscopia, e a micromola foi facilmente localizada por meio de palpação pulmonar através de uma minitoracotomia de 3 cm (Figura 1E). O diagnóstico final foi de adenocarcinoma pulmonar. 

**Figura 1:** Em A, imagem de TC mostrando um nódulo de 1 cm em vidro fosco na porção central do lobo superior direito. Em B, reconstrução tridimensional de uma imagem de TC mostrando a agulha dentro do nódulo em vidro fosco no lobo superior direito. Em C, reconstrução tridimensional de uma imagem de TC mostrando a colocação, guiada por TC (seta), de uma micromola de platina no interior do nódulo em vidro fosco. Em D, imagem de TC obtida após o procedimento, mostrando a micromola de platina (seta) no interior do nódulo em vidro fosco. Em E, fotografia da peça cirúrgica mostrando a micromola.

As técnicas para a localização de nódulos pulmonares foram classificadas em três tipos. ^(^ 1 ^,^ 4 ^)^ A primeira classe emprega exames de imagem intraoperatórios (ultrassonografia ou TC). A localização por meio de ultrassonografia intraoperatória é difícil porque o pulmão deve estar em completo colapso para que se possam visualizar nódulos pequenos.^(^ 1 ^,^ 4 ^)^ Essa técnica aumenta o tempo cirúrgico, pois o colapso total do pulmão pode levar de 30 min a 150 min e é frequentemente contraindicado em pacientes com enfisema extenso. Além de a experiência com resseção toracoscópica guiada por TC em tempo real ser limitada, artefatos causados por instrumentos e grampos degradam a imagem da TC, e o espaço limitado dentro do pórtico (*gantry*) do tomógrafo torna o procedimento difícil.^(^ 1 ^,^ 4 ^)^

A segunda classe de técnicas de localização inclui a injeção percutânea de corantes, meios de contraste, radionuclídeos ou agentes adesivos coloridos.^(^ 1 ^,^ 4 ^,^ 5 ^)^ A difusão longe do nódulo é uma limitação dessas técnicas e impõe restrições sobre o tempo admissível entre o procedimento de localização por TC e a resseção toracoscópica. Isso pode causar dificuldades na programação do centro cirúrgico. Além disso, há risco de reações anafiláticas após a injeção de certos corantes, como o azul de metileno, e é muitas vezes difícil visualizá-los na superfície pleural visceral em pacientes com extensa pigmentação antracótica dos pulmões.^(^ 1 ^,^ 4 ^,^ 5 ^)^ Como esses materiais não são solúveis em água, há risco de acidente vascular cerebral caso cheguem às veias pulmonares. 

A terceira classe de técnicas de localização emprega molas ou micromolas que são macias e flexíveis e causam pouco dano ao tecido pulmonar, mesmo quando se desalojam. Um estudo anterior comparou micromolas e fios em forma de gancho quanto a seu desempenho na localização de nódulos em pulmões de cabra recém-colhidos.^(^ 5 ^)^ Os autores relataram que a micromola se desenrolava sempre que se deslocava, causando dano mínimo ao tecido. Além disso, o revestimento "felpudo" de fibra dessas micromolas induz a coagulação e aumenta a aderência da micromola ao tecido pulmonar. A configuração espiral e o revestimento de fibras praticamente eliminam o risco de embolização. 

Em suma, gostaríamos de destacar esse novo método de localização de nódulos, que se trata de uma técnica segura e eficaz e que aumenta a taxa de sucesso de excisão de nódulos por meio de videotoracoscopia, especialmente em casos de pequenos nódulos em vidro fosco.
